# Biosynthesis and Characterization of Nanocellulose-Gelatin Films

**DOI:** 10.3390/ma6030782

**Published:** 2013-02-28

**Authors:** Siriporn Taokaew, Sutasinee Seetabhawang, Pongpun Siripong, Muenduen Phisalaphong

**Affiliations:** 1 Chemical Engineering Research Unit for Value Adding of Bioresources, Department of Chemical Engineering, Faculty of Engineering, Chulalongkorn University, Bangkok 10330, Thailand; E-Mails: orangesom40@hotmail.com (S.T.); sutasineeseeta@yahoo.com (S.S.); 2 Natural Products Research Section, Research Division, National Cancer Institute of Thailand, Bangkok 10400, Thailand; E-Mail: pongpun@health.moph.go.th

**Keywords:** bacterial cellulose, gelatin, nanofibril, film

## Abstract

A nanocellulose-gelatin (bacterial cellulose gelatin (BCG)) film was developed by a supplement of gelatin, at a concentration of 1%–10% w/v, in a coconut-water medium under the static cultivation of *Acetobacter xylinum*. The two polymers exhibited a certain degree of miscibility. The BCG film displayed dense and uniform homogeneous structures. The Fourier transform infrared spectroscopy (FTIR) results demonstrated interactions between the cellulose and gelatin. Incorporation of gelatin into a cellulose nanofiber network resulted in significantly improved optical transparency and water absorption capacity of the films. A significant drop in the mechanical strengths and a decrease in the porosity of the film were observed when the supplement of gelatin was more than 3% (w/v). The BCG films showed no cytotoxicity against Vero cells.

## 1. Introduction

Advances in the field of biomaterials have generated a wide range of materials of interest. Among them, cellulose has been used as a biomaterial for many applications [1]. Cellulose, hemicellulose and lignin are the major components of plant skeletal polysaccharides. Pure nanocellulose can be produced by the bacterium *Acetobacter xylinum* via fermentation [2,3,4]. Bacterial cellulose (BC) has the same chemical structure as plant cellulose, but has an ultrafine nanofiber network structure and unique properties, including high crystallinity, high water holding capacity, high tensile strength, high purity and flexibility [5,6]. It has been reported that BC has potential uses in food, cosmetics and medical applications. BC has been developed as artificial skin for humans with extensive burns [7], artificial blood vessels for microsurgery [8], scaffolds for tissue engineering of cartilage [9] and wound-dressing [10]. Innovative BC films for food, cosmetic and medical applications have been developed concurrently with a wide range of supplemental candidate materials, such as alginate, polyurethane, chitosan and gelatin. 

Gelatin is a natural protein polymer that is derived from the hydrolysis of collagen and possesses an average molecular weight between 65,000 and 300,000 g/mol [11]. Gelatin has a relatively low antigenicity compared to collagen, but it still retains some of the informational signals that may promote cell adhesion, differentiation and proliferation. Because of its excellent biodegradability and biocompatibility, many recently studied biomaterials are gelatin based, and applications of these include artificial skin, wound dressing, bone grafts, plasma expander, scaffolds for tissue engineering, adhesives and absorbent pads [12,13,14]. Gelatin gels are transparent, flexible and easily soluble in hot water. In their dry state, gelatin films have a typical brittle behavior [15], which limits their application.

From our previous work, it can be noted that physical and biological properties of BC can be modified by supplementing the BC culture medium with low-molecular-weight chitosan [16,17] and alginate [18]. Modifications of BC properties by incorporating other composites to further enhance its biocompatibility have been reported. Collagen-BC composites prepared by adding collagen type I to the BC culture medium are reported to be able to reduce the amount of selected proteases and interleukins significantly and possess a distinct antioxidant capacity for the treatment of chronic wounds [19]. Trimethyl ammonium betahydroxy propyl-BC (TMAHP-BC), covered with adhesive proteins (collagen type I, collagen type IV, fibrin, fibronectin or laminin), was developed to promote endothelial cell adhesion and metabolism [20,21]. Several syntheses and characterizations of blend membranes of cellulose with gelatin, collagen and other biopolymers have been reported [21]. To improve the positive features of BC, BC-gelatin composites prepared by immersion and crosslinking have been developed and characterized [22,23,24]. However, very few, if any, studies of biosynthesis of BC-gelatin films have been reported.

This study aims to prepare composite films that combine the advantageous properties of gelatin with the excellent biological and physical properties of nano-bacterial cellulose by means of adding gelatin in the culture medium under static cultivation of *Acetobacter xylinum*. For further applications, important physical, chemical and biological properties were characterized. 

## 2. Results and Discussion

### 2.1. Morphology

**Figure 1:**
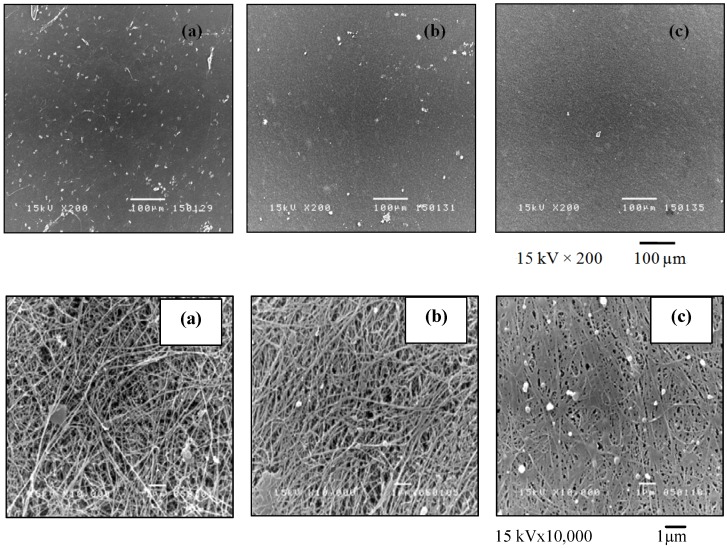
The overview (top) and high magnification (bottom) photomicrographs of the bacterial cellulose (BC) and bacterial cellulose gelatin (BCG) films: (**a**) BC; (**b**) BCG-3 and (**c**) BCG-10.

**Figure 2:**
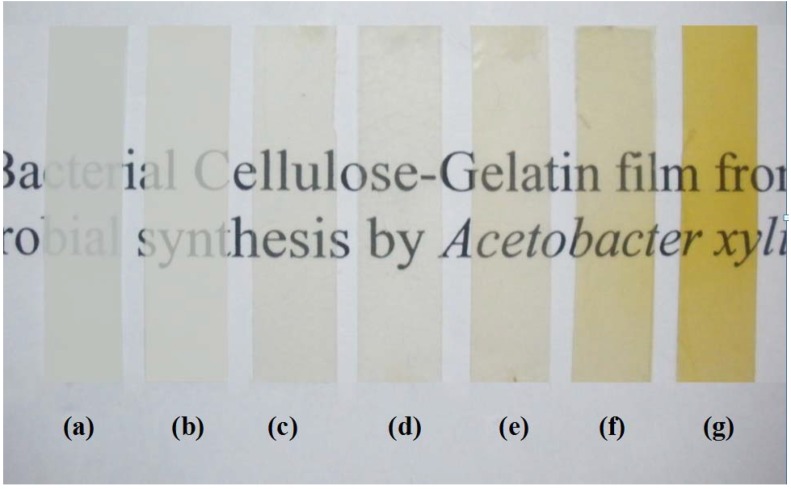
Optical photographs of the BC, BCG and gelatin films: (**a**) BC; (**b**) BCG-1; (**c**) BCG-3; (**d**) BCG-5; (**e**) BCG-7; (**f**) BCG-10 and (**g**) gelatin.

The surface structures of BC and bacterial cellulose gelatin (BCG) films were analyzed by scanning electron microscopy (SEM). The overview and high magnification photomicrographs of the film surface are shown in Figure 1. The BC and BCG films refer to the cellulose films without and with the addition of gelatin in the culture medium, respectively, whereas, BCG-*x* refers to BCG with *x*% (w/v) of gelatin addition. The fiber of the BC films was approximately 50–80 nm in diameter. By adding gelatin in the culture medium, gelatin was well incorporated into the cellulose fibril network and filled the pores. During the purification of the modified BC sheet, some gelatin softgels on the sheet surface was observed. The structure of the BCG film was denser, with a smaller pore size than that of the BC film. With the addition of gelatin at 10% (w/v) in the culture medium, the gelatin gel almost completely filled the space of the BC sheets. The incorporation of gel into the network was similar to the alginate gel filling in the BC fibril network described in our previous work [18]. Figure 2 illustrates the effect of the concentration of gelatin on the transparency of the BCG film. It was clearly observed that the incorporation of gelatin in the BCG films between 3% and 10% (w/v) resulted in a considerable improvement in optical transparency of the films. According to previous reports [25,26], certain polymers, such as epoxy and acrylic resins, poly (ethylene glycol) and poly (vinyl alcohol), could fill in the empty space between BC fibrils while retaining the optical transparency of each polymer. The transparency of the film for wound dressing is of considerable importance, as it would permit clinical observation of the wound area and of the healing process without removal of the wound dressing [27]. Transparency is also an important property of films used for food packaging. 

### 2.2. FTIR Analysis 

**Figure 3:**
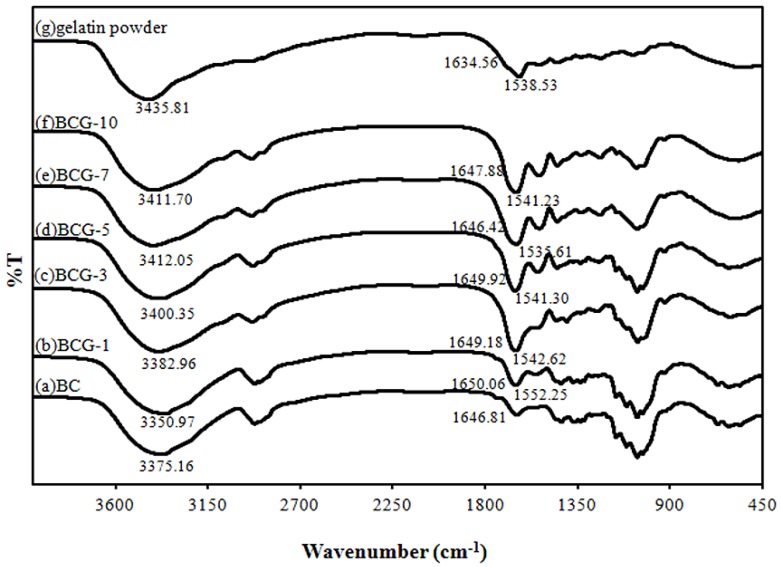
The Fourier transform infrared spectroscopy (FTIR) spectra of the BC, BCG and gelatin films: (**a**) BC; (**b**) BCG-1; (**c**) BCG-3; (**d**) BCG-5; (**e**) BCG-7; (**f**) BCG-10 and **(g)** gelatin powder.

The Fourier transform infrared spectroscopy (FTIR) spectra of all samples were detected at wave numbers ranging from 3600 to 900 cm^−1^ (Figure 3). 

The FTIR spectra show strong absorption in the range of 1646–1650 cm^−1^, which demonstrates the presence of a carbonyl group (–COO) in BC, whereas absorption in the range of 3350–3412 cm^−1^ demonstrates the occurrence of hydroxyl groups [16,18,28]. The characteristic absorption bands of the gelatin film were the bands at 3299.60, 1631.10 and 1529.82 cm^−1^, which were identified as the O–H stretching vibration, amide I (C=O and C–N stretching vibration) and amide II (N–H bending vibration), respectively [12,29]. The FTIR results demonstrate that the characteristic absorption bands of all the BCG films, which were supplemented with 1%–10% w/v gelatin in the culture medium, are slightly shifted from a peak at 1646.81 cm^−1^ to a peak between 1650.06 and 1647.88 cm^−1^. The BCG films also show a shift of the amide II peak to 1552.25–1541.23 cm^−1^, due to the presence of the N–H bending vibration of gelatin. The observed peak at 3350.97–3411.70 cm^−1^ of the BCG films was attributed to O–H stretching vibrations, and they were shifted compared to the peaks observed on the spectra of the BC (3375.16 cm^−1^) and gelatin films (3435.81 cm^−1^). The frequency difference could be considered as a measure of the average strength of the intermolecular hydrogen bonds [30,31]. All of the peak changes could imply some specified interactions between the characteristic groups of BC and gelatin, leading to a good molecular compatibility between BC and gelatin.

### 2.3. Crystallinity 

**Figure 4:**
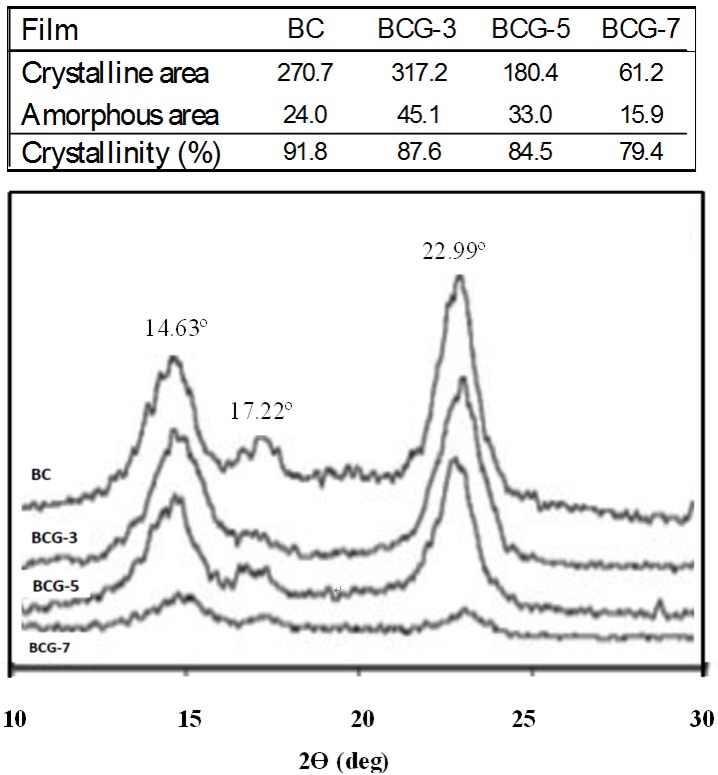
The X-ray diffractometer (XRD) patterns and crystallinity (%) of the BC and BCG films: (**a**) BC; (**b**) BCG-3; (**c**) BCG-5 and (**d**) BCG-7.

In this study, a X-ray diffractometer (XRD) was used to determine the crystallinity of the films. The XRD patterns and crystallinity (%) of BC and BCG films are shown in Figure 4. Three main peaks were present at 14.63, 17.22 and 22.99 deg for the (110), (110) and (200) planes, respectively, and they can be identified as the reflexion planes of cellulose I [29]. These observed peaks were attributed to the BC cultured in static circumstances [16]. Generally, gelatin has a broad diffraction peak in the 2θ range between 20 and 22 deg. For the BCG films, there was no observation of a gelatin diffraction peak. From this, we infer that the crystallinity of the films was mainly due to BC. It was shown that the adding of gelatin at 7% (w/v) in the culture medium significantly reduced the crystallinity of the films, as shown in the line broadening of the crystalline cellulose. A probable cause of this could be the increased viscosity of culture medium from the gelatin supplement, which resulted in a lower speed of motion of *Acetobacter* during the cellulose synthesis and would consequently alter the BC structure. The viscosity of the culture medium without adding alginate was 8.8 centipoise (cP). With the addition of alginate at 3%, 5% and 7% (w/v) in the culture medium, the viscosity of the culture medium was increased to 18.4, 24.8 and 36 cP, respectively. During the cultivation, significantly slower rates of BC pellicle formation in the culture medium with the addition of alginate of more than 5% (w/v) were observed. The addition of alginate of more than 10% (w/v) strongly inhibited the formation of BC pellicle. Therefore, the study of gelatin supplement in the culture medium was performed in the concentration range of 0%–10% (w/v). In our preliminary study, we observed no significant change in the crystalline structure of BC-gelatin films prepared by immersing the wet BC pellicle in 0%–30% (w/v) gelatin aqueous solutions (data not shown). The similar result was reported by Kim* et al.* in 2010 [29]. However, in this study, the modification by adding gelatin in the culture medium showed the influence on crystallinity of the films. With increasing the culture medium viscosity, the oxygen transfer rate and cell motion decreased. Therefore, the plausible explanation is that the changed conditions and behavior of cells during BC biosynthesis might alter the cellulose microfibril structure, resulting in reduction of crystallinity. It was reported earlier that growing bacteria in a viscous medium [32] or at low temperature [33] affected bacterial motion and, consequently, affected the rate of cell division [34]. It was also reported that the supplement of some agents during microbial synthesis could affect the assembly and crystallization of glucan chains, as the motion of bacterium was changed [35]. *In vivo* cellulose ribbon assembly by the Gram-negative bacterium, *Acetobacter xylinum*, could be altered by incubation in carboxymethyl cellulose (CMC) and also by incubation in a variety of neutral, water-soluble cellulose derivatives [36]. However, no change in crystallinity was observed, but when an optical brightener was used, the crystallinity was significantly reduced. Perhaps the addition of gelatin during synthesis could interfere with the final assembly of the glucan chain aggregates as they are emerging from the pores of the bacterial cell. This might also indicate a greater affinity of the gelatin for cellulose than CMC.

### 2.4. Mechanical Property

**Figure 5:**
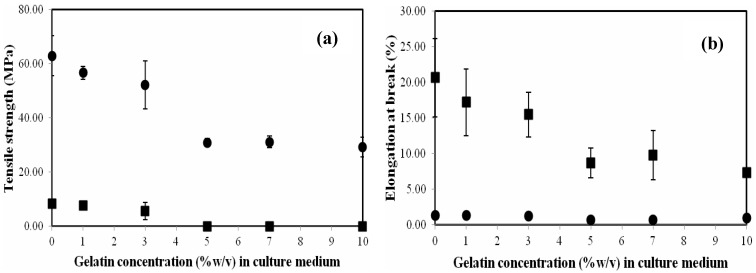
The tensile strength (**a**) and the elongation at break (**b**) of the BC and BCG films as a function of gelatin concentration in culture medium: dried film (●); reswollen film (■).

The tensile strength and elongation at the break point of the films with an average thickness of 0.1 mm are shown in Figure 5a,b, respectively. The average tensile strength and the average elongation at the break point of dried BC films were 63.0 MPa and 1.4%, respectively. After the films were immersed and equilibrated in deionized water (DI) water, these values became 8.3 MPa and 20.7%, respectively. The strength of the BC film in reswollen form was lower, but it exhibited more elastic behavior than that of the BC film in dried form. The tensile strength of BCG films in dried and reswollen forms slightly decreased with the addition of gelatin at a concentration of 1%–3% (w/v). However, a considerable decline of the mechanical strength with a supplement of gelatin at a concentration greater than 3% (w/v) was observed. The tensile strength of the BCG films in dried and reswollen forms significantly decreased to approximately 29.3–32.1 and 0.02–0.03 MPa, respectively, with the addition of gelatin at a concentration of 5%–10% (w/v). Similar observations in tensile strength reduction in BC films modified with biopolymers, such as polyethylene glycol (PEG) [28] and alginate [18] were previously reported. The plausible explanation for the observed behavior is that, at low gelatin concentrations (<5% w/v), the cellulose fibrils are only partially coated with gelatin. During dehydration, hydrogen bonding between cellulose fibrils is still possible, which results in a material with higher tensile strength. At 5% w/v gelatin supplement, the cellulose fibers are fully coated with gelatin. After dehydration, the bonding between fibers is through the gelatin coating and the bound gelatin-gelatin interactions dominate the mechanical properties. After rehydration, the composites with less than 5% w/v gelatin supplement are stronger, because the cellulose-cellulose hydrogen bonding is more stable (less disrupted by water) than gelatin-gelatin bonding. Gelatin is soluble, so composites with ≥5% w/v gelatin supplement (coated cellulose fibers) have little mechanical strength. It is well known that thermally dehydrated BC cannot be returned to its never-dried state by rehydrating. The collapse of the cellulose fiber network is not reversible. As shown in Figure 5b, the elongation at the breaking point of the BCG films from the addition of 5%–10% (w/v) gelatin was also significantly reduced to 0.8%–0.9% (in dried form) and 7.3%–9.8% (in re-swollen form) or approximately half of the respective values of the BC film. It was previously reported that the films modified with gelatin were more brittle due to the brittleness of gelatin [37]. Nevertheless, after the BCG films were re-swollen in water; they became more elastic due to the swelling of the cellulose fiber and gelatin in water. 

### 2.5. Water Absorption Capacity (WAC)

**Figure 6:**
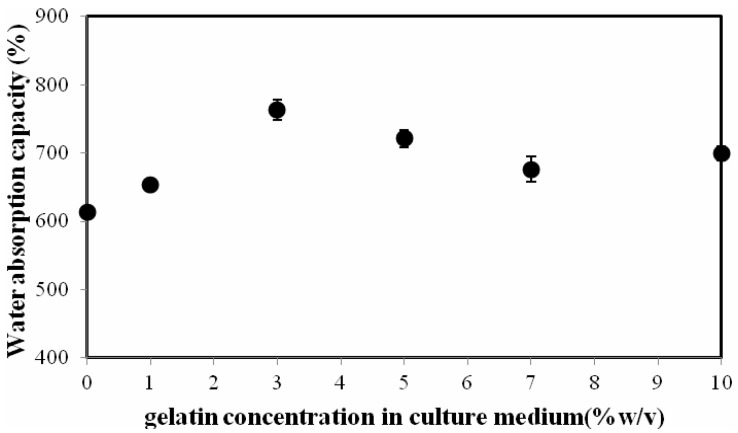
The water absorption capacity of the BC and BCG films as a function of gelatin concentration in culture medium.

As shown in Figure 6, the Water Absorption Capacity (WAC) of the BCG films increased with increases in the gelatin content up to a concentration of 3% (w/v) and slightly decreased with the further addition of gelatin. The WAC values of the BC and BCG-3 films were 613.8% and 762.5%, respectively. The film modified with gelatin exhibited the greater WAC due to the high hydrophilic character of gelatin. However, with excessive gelatin addition, the film structure became miscellaneous and weak; consequently, the WAC of the film decreased. Similar observations were previously reported from BC films modified with aloe vera [38].

### 2.6. Gas (Vapor) Transmission Rate 

The water vapor transmission rate (WVTR) of the BCG-3, BCG-5 and BCG-10 films ranged between 592 and 885 g/m^2^/day, which was to some extent lower than that of the BC film (1026 g/m^2^/day), due to the denser film structure. The WVTR of modified BC films slightly decreased as a result of the denser structure, and a decrease in pore size from the supplement of alginate was previously reported [18]. The oxygen transmission rate (OTR) values of the BC and BCG films were all in a similar range (between 1.6 and 2.7 cc/m^2^/day). Due to the different drying conditions, the OTR and WVTR of the BC films prepared in this study were relatively lower than those from previous work [18,38].

### 2.7. Cytoxicity

The cytotoxicity of the BCG films was tested on the Vero cell line. The absorbance of living Vero cells after seeding on the BCG-10 films for 0, 24 and 48 h was compared to that of the cells cultured on the BC films (control), as shown in Table 1. The BCG and BC films showed no cytotoxicity against Vero cells. The BCG-10 films could support the cell proliferation to the same extent compared with the BC films. BC has been previously reported as a non-pyrogenic, non-toxic and fully biocompatible material for tissue engineering and wound healing [10].

**Table 1:** Cell viability results from MTT assay of Vero cells on BC (control) and BCG-10 films.

Type of films	Absorbance		
	0 h	24 h	48 h
BC	0.298 ± 0.037	0.376 ± 0.029	0.481 ± 0.036
BCG-10	0.256 ± 0.020	0.342 ± 0.044	0.589 ± 0.052

## 3. Experimental Section 

### 3.1. Microbial Strains

*Acetobacter xylinum*, AGR60, was isolated from nata de coco. The stock culture was kindly supplied by Pramote Thammarad, the Institute of Research and Development of Food Product, Kasetsart University, Bangkok, Thailand.

### 3.2. Culture Media and Method

The medium for the inoculum was coconut water that contained 5.0% sucrose, 0.5% ammonium sulfate and 1.0% acetic acid. The medium was sterilized at 110 °C for 5 min. Pre-cultures were prepared by transferring 10 mL of a stock culture to 200 mL of medium in 500 mL bottles, followed by static incubation at 30 °C for 7 days. After the surface pellicle was removed, the 5% (v/v) pre-culture broth was added to the main culture medium and supplemented with different contents of gelatin (Type A, 300 bloom, Sigma Chemical, St Louis, MO, USA). To prepare gelatin solution, the gelatin powder was added in the DI water at room temperature (30 °C) and the gelatin solution was heated at 50 °C and stirred for about 10 h to completely solubilize the gelatin. After the gelatin solution became clear and colorless to light yellow, it was added to the culture medium. The 75 mL of the culture medium or modified culture medium, mixed with the pre-culture broth, was then inoculated in a 14.5 cm diameter Petri-dish and kept at 30 °C for 7–10 days. The developed gel-like cellulose pellicle was then purified by washing with DI water for 1 h and subsequently treated with 1% (w/v) NaOH at room temperature for 24 h to remove bacterial cells. This was followed by a rinse with DI water until the pH reached 7.0. Afterwards, the purified BCG sheet was vacuum-dried at 40 °C for 3 h and then air-dried at room temperature (30 °C) for 2 days. All dried films were stored in plastic films at room temperature before use.

### 3.3. Characterizations

The surface properties were examined with a JOEL JSM-5410LV scanning electron microscope (SEM) (Tokyo, Japan). The films, in re-swollen form, were dehydrated in ascending grades of ethanol and dried in a Tousimis Samdri-780 critical point dryer (Maryland, USA) using liquid carbon dioxide as a transitional fluid. The dried films were sputtered with gold in a Balzers-SCD 040 sputter coater (Balzers, Liechtenstein). The accelerating voltage was adjusted to 15 kV. The specimens were examined at a magnification of 10,000×. 

FTIR spectroscopy is used primarily to identify the chemical structure of the sample. FTIR spectra of the developed films were recorded with a Perkin Elmer FTIR (Spectrum One, Massachusetts, USA) for a range of wavenumbers between 3600 and 1000 cm^−1^.

Crystallinity was measured with an X-ray diffractometer (Model D8 Discover, Bruker AXS, Karlsruhe, Germany). X-ray diffraction patterns were recorded with CuKa radiation (k = 1.54 Å). The operating voltage and current were 40 kV and 30 mA, respectively. Samples were scanned from 10–40° 2θ at a scan speed of 3° min^−1^. Profile fitting and crystallinity (%) calculations were performed with Topas version 3.0 (Bruker, AXS) software.

Mechanical property was measured by Instron Testing Instron (5567, NY, USA). The film samples were cut into strip-shaped specimens with a width of 10 mm and a length of 10 cm. The test conditions follow ASTM D882 guidelines. The tensile strength and break strain were reported as average values determined from at least five specimens. 

Water absorption capacity (WAC) was determined by immersing previously weighed films in DI water at room temperature (30 °C) until they were equilibrated. After that, the films were then removed from the water, and excess water at the surface of the films was blotted out with Kimwipes^®^. The weight of the swollen films was measured, and the procedure was repeated until there was no further weight change. Water content was calculated using the following formula:

$$WAC(\%)=\frac{W_{h}-W_{d}}{W_{d}}\times 100$$

where *W*_h_ and *W*_d_ denote the weights of the hydrated and dry film.

The gas (vapor) transmission rate of the dry film with an area of 50 cm^2^ was determined at the Thai Packaging Centre, Thailand Institute of Scientific and Technological Research (Bangkok, Thailand). The oxygen transmission rate (OTR) was determined with an oxygen permeation analyzer (Model 8000, Illinois Instruments, Johnsburg, IL). The test conditions followed ASTM D-3985 guidelines. The OTR was determined under 23 °C and 0% relative humidity. The test conditions for water vapor transmission rate (WVTR) followed ASTM E-96 with desiccant method guidelines. The WVTR was determined under 38 °C and 98% relative humidity. 

Cytotoxicity evaluation of the films was conducted with Vero cells, which were isolated from African green monkey’s kidney cells (Vero, ATCC CCL-81). The dried BCG films were punched into round-shaped samples that were 16 mm in diameter. The samples were sterilized by autoclaving at 121 °C for 10 min and were then transferred aseptically to 24-well culture plates. The experiments were conducted in triplicate. One milliliter of minimum essential medium (MEM) culture medium with 10% fetal calf serum was added to each well and allowed to equilibrate with the samples for 30 min and then removed. A metallic ring and 0.5 mL of MEM culture medium with 10% fetal calf serum were added to each well before cell seeding. Vero cells were seeded onto the 24-well culture plates at an initial density of 6 × 10^4^ cells per well on both the BCG film and the control. Cells were incubated at 37 °C in a humidified atmosphere containing 5% CO_2_ for 16 h. Then, the culture medium was exchanged with serum-free MEM for another 48 h for the culture of Vero cells. At the end of incubation, the number of living cells was determined using the MTT assay. 

## 4. Conclusions 

The supplementation of gelatin in culture medium during BC biosynthesis could modify the morphology and properties of the films. Gelatin was well incorporated into the cellulose fibril network and filled the pores; the BCG film was denser than the BC films. The films exhibited a lower crystalline order upon supplementation with gelatin. FTIR spectroscopy indicated that there might be interactions between the glucose carbonyl groups of the BC fiber and the amine groups of the gelatin. The incorporation of gelatin in the BCG films resulted in a significant improvement in the optical transparency of the films. With the addition of gelatin, up to 3% (w/v), the BCG films showed higher degrees of swelling and exhibited more hydrophilic characteristics, resulting in improved water absorption capacity. However, the addition of gelatin at a concentration greater than 3% (w/v) resulted in a significant decrease in tensile strength and elongation at the breaking point of the BCG films. The cytotoxicity test on the Vero cell line showed that BC and BCG were non-toxic and biocompatible materials.
